# Memoir upon the Muriatic Ether, Lately Read at the Institute of France

**Published:** 1808-08

**Authors:** M. Thenard


### *Memoir upon the Muriatic Ether, lately read at the Institute of France.* By M. Thenard.†

AFTER having examined why the muriatic either has hitherto remained almost unknown among chemists, although frequently the object of chemical experiments, the author gives the method of obtaining it. For this purpose, as the above ether is habitually in the state of gas, the following apparatus must be employed.

We

---

† *Ann, de Chimie,* tom. lxi. p. 290.

We put in a retort barely capable of containing the mixture, an equal part in volume of highly concentrated muriatic acid and alcohol at 36°; we shake them well, in order to bring all their molecules in contact. This being done, we throw into the retort seven or eight grains of sand, in order to avoid the boiling up, which, without this precaution, might take place in the course of the operation; we then place it in the naked fire upon a common furnace, by means of a grating of iron wire, and adopt Welter's tube to it, which enters into a flask with three necks, double in capacity to the retort employed, and half filled with water at the temperature of 20 or 25°,* so that the tube penetrates a considerable way into the water: afterwards we introduce into the second neck a straight tube of safety; and into the third we introduce a crooked one, which is fixed into an earthen vessel under flasks full of water, at the same degree of heat as the former, and supported by a knob screwed into the middle of it. When the apparatus is thus arranged, the retort must be gradually heated; and in about 20 or 25 minutes we see bubbles arise from the lower part of the liquid, and particularly from the surface of the grains of sand. These bubbles soon increase, and abundance of etherized gas is obtained; acid, alcohol, and water, pass over at the same time, but they remain in the first flask. From 500 grammes of air, and an equal volume of alcohol, we may obtain twenty litres and upwards of etherized gas perfectly pure. But we shall extract much more from it, if, when the extrication of the gas begins to slacken, we add a fresh quantity to the residue, namely, the strongly acid liquor which remains in the retort, and the volume of which is then nearly equivalent to two-fifths of the liquor from which it comes. M. Thenard even thinks, that if, by means of a straight tube going to the bottom of the retort and of a proper length, we could pour from time to time warm alcohol into the latter, the etherized gas would be formed in still greater abundance; for we should conceive that there is every moment more alcohol volatilized than muriatic acid, and that we should thus re-establish between these two bodies the primitive proportions, which are more proper than any other for the success of the operation. In all cases the management of the fire is of the greatest importance: if it be too weak, no etherized gas is produced; if too strong, but very little is produced. Besides, we do not

* The centigrade thermometer is the one intended.

not etherize the alcohol sensibly by charging it with muriatic acid gas, nor do we obtain ether more sensibly by bringing together the acid and the alcohol in vapours into a tube about the temperature of 80°. It is therefore by preserving a just medium in the application of the fire that we succeed completely. All this proceeds from too small or too great an elasticity in the alcohol, and the muriatic acid prevents their re-action upon each other. One precaution we must also take, is to use the same water for collecting the gas, and to employ the least quantity possible, because it dissolves it in a remarkable degree.

This gas is absolutely colourless; the smell of it is strongly etherized, and the taste sensibly saccharine. It has no kind of action either upon turnsole tincture, syrup of violets, or lime-water. Its specific gravity compared to that of air is 2·219 to + 18° of the centigrade thermometer, and at $0^m$ 75 of pressure at the same temperature, and at the same pressure water dissolves its own volume of it. At this same degree of pressure also, but at + 11° of temperature, the etherized gas becomes liquid. We may procure a great quantity of it in this state, by using an apparatus similar to that we have just described; simply, in place of fixing the last tube under a flask full of water, we must plunge it to the bottom of a long, straight, well-dried probe, and surrounded with ice, which we must renew in proportion as it melts. It is in this probe that the etherized gas alone arrives and is entirely liquefied; for, when once the vessels contain no more air, we may, without the least danger, suppress its communication with the atmosphere.

When thus liquefied, this ether is of a remarkable limpidity, as in the state of gas it is without colour and without action upon turnsole tincture and syrup of violets; as well as the etherized gas, it is very soluble in alcohol, from which we may, in a great measure, separate it by water: like this gas, it has also a very decided smell, and a very distinct taste, which has something analogous to that of sugar, and which is particularly remarkable in water which is saturated with it, which may, perhaps, be employed successfully in medicine. When poured upon the hand, it suddenly evaporates, and produces a considerable cold, leaving a small whitish residue. At + 5° of temperature, (centigrade thermometer,) it weighs 874, water weighing 1000. Thus, although it is far more volatile than the sulphuric ether, and, *a fortiori*, than alcohol, not only is it thicker than the former, but even than the latter of these two bodies. Lastly, it does not congeal at a temperature of — 29° (centigrade thermometer.)

Hitherto

Hitherto we have seen nothing in this ether which does not perfectly agree with that presented by other matters; it is nothing else than a substance curious from its novelty, and particularly from the facility with which it is gasified and liquefied. When we reflect upon it a little more, it appears one of the most singular and extraordinary compounds we can produce. It does not in the least redden turnsole tincture; the strongest alkalis have no action upon it; the solution of silver does not meddle with it at all; and all this, whether we employ it in the gaseous or liquid state, or dissolved in water: if we set fire to it, there is suddenly developed such a quantity of muriatic acid, that this acid precipitates in a mass the concentrated nitrate of silver, suffocates those who respire it, and even appears in the form of vapours in the surrounding air.

Is the muriatic acid formed in this inflammation, as we are induced to think, or is it only set at liberty? This is the question which the author of the memoir afterwards endeavours to resolve.

If the muriatic acid be formed in the combustion of the etherized gas, the radical of this acid must exist in the gas; and this radical necessarily comes from the alcohol, or from the muriatic acid decomposed by the alcohol, or, what is not very probable, although not impossible, from both. In the first case, by distilling a mixture of alcohol and muriatic acid, we ought to find after the distillation all the muriatic acid employed, besides that which appeared in the combustion of the gas formed; in the second case, on the contrary, a great quantity of acid ought to disappear in this distillation; but by keeping an account of that which is developed in the combustion of the gas formed, this quantity of acid precisely, and no more, ought to re-appear entirely. In the third case, from this distillation, a loss of acid should also result; but this loss should be more than compensated by the quantity of acid which the combustion of the gas formed ought to produce. Now, on performing this distillation upon 450·937 grammes of muriatic acid of a specific gravity of 11·349, at 5° temperature (centigrade thermometer,) and upon a volume of highly rectified alcohol equal to that of the acid, there are formed 23 litres of etherized gas at the temperature of 21° of the centigrade thermometer, and at the pressure of $0^{m}$ 745, and there disappear 122·288 grammes of acid.

The first hypothesis is consequently false, since it is demonstrated that, even should the radical of the muriatic acid exist in the etherised gas, this radical would proceed not not merely from the alcohol, but rather either from the muriatic acid alone, or from the muriatic acid and alcohol.

Let us inquire if it proceeds from the muriatic acid alone, as supposed in the second hypothesis: but here there are two ways of considering the phænomenon: either the muriatic acid must have been decomposed by the alcohol, so that its radical, without its other principle, is to be found in the etherized gas; or this decomposition will have been such, that all the principles of the muriatic acid will be found in the etherized gas, not united, and not forming muriatic acid, but combined with the principles of the alcohol, and in the same state in which hydrogen, oxygen, carbon, and azot exist in animal or vegetable matters. Now if the radical of the muriatic acid exists alone in the other principle, or without a portion of the other principle of the muriatic acid in the etherized gas, we ought, by decomposing this gas in a red-hot tube, and deprived of the contact of the air, to obtain no acid at all, or else less of it than has disappeared in the experiment which produced it: and if this gas contains not only the radical of the muriatic acid, but also all the constituent principles of that acid; as those principles, whatever they are, have a strong tendency to combine, we should conceive that by destroying the etherized gas by fire, without the contact of the air, we shall probably obtain the whole quantity of muriatic acid which has disappeared in the experiment by which we procured it. It was therefore of the greatest importance to produce this decomposition in close vessels. The operation was performed on 900 grammes of concentrated muriatic acid, and upon an equal volume of well-rectified alcohol. Between the red-hot glass tube, in which the gas was decomposed, and the retort in which it was produced, there was a large tubulated flask containing water at about 15° or 16°, in order to catch the acid, the alcohol and the water, which might be volatilized along with this gas; the glass tube, besides, communicated with two other flasks, one containing potash and the other water, in order to absorb all the acid which might reappear in the operation: finally, the gases were collected by means of another tube. To ensure the success of the operation, the glass tube should be well luted, and the fire also well managed to prevent the tube from melting. Although in this experiment there ought to have been produced nearly 50 litres of etherized gas, and nearly 250 grammes of acid ought to have disappeared in the first place, yet all the acid except four grammes re-appeared in the red-hot tube, and came to be dissolved in the two flasks of the apparatus.

Thus,

Thus, of all the suppositions hitherto formed of the muriatic acid being a compound body, one only is admissible, which infers that the elements of the muriatic acid exist in the etherized gas combined with the elements of the alcohol, in the same manner as the elements of water, carbonic acid, ammonia, &c. exist in vegetable and animal substances.

Now if we suppose the muriatic acid to be a simple body, we must then necessarily regard etherized gas as formed of muriatic acid and alcohol, or of a body coming from the decomposition of alcohol; for alcohol is perhaps decomposed when we distil it with the muriatic acid. In all cases, the question is thus brought to a choice of the two hypotheses. Let us now try their merits as well as we can.

One, namely that which we have last mentioned, presents us with phænomena of difficult explanation. In fact, it must be supposed that alcohol, or the body which represents it, acts upon the muriatic acid with much more energy than the strongest alkali, since this alkali cannot take it off from it, and since, as will be subsequently demonstrated, the muriate of potash contains less acid than the etherized gas; and how can we conceive, on the other hand, that the nitrate of silver, which takes up all the muriatic acid from the muriate of potash, cannot take it up from the etherized gas, which contains more than the muriate of potash does?

In the other hypothesis, on the contrary, every thing is naturally explained. We see the reason why the etherized gas does not redden turnsole tincture, and the alkalis do not alter it; why the nitrate of silver produces no precipitate from it; and why, on taking fire, so large a quantity of muriatic acid is produced, that it appears in the air in the form of vapours:—in short, we can reconcile every thing with the phænomena of other bodies.

M. Thenard, however, is far from admitting the one and rejecting the other in a decided manner. Both deserve to be investigated; and whatever happens, the results must be very important.

---

### *Note upon the Discovery of the Muriatic Ether.*
By M. Thenard.*

When I read the foregoing memoir to the Institute, the whole of the members, among whom were Messrs. Berthollet,

* *Ann. de Chimie*, tom. lxi. p. 303.

thollet, Chaptal, Devoux, Fourcroy, Guyton, Vauquelin, Gay-Lussac, &c. regarded the results it contained as being extremely novel, and were struck with the consequences which might be drawn from it. M. Proust, who was then in Paris, and before whom I repeated the experiment of testing the etherized gas by the tincture of turnsole and the nitrate of silver, before and after the combustion of the gas, &c. evinced the same surprise with the French chemists. A few days afterwards, however, M. Gay-Lussac, on perusing the German Journal of Gehlen, accidentally discovered in a note that Gehlen himself had made experiments upon the muriatic ether, and published them in his Journal for 1804. M. Gay-Lussac did me the favour to translate M. Gehlen's memoir; and as it has a great similarity to mine, I shall give the following extract.

M. Gehlen made muriatic ether with the smoking muriate of tin and alcohol, by employing an equal part in weight of both. He also made it in Basse's method, (a chemist of Hameln) by a mixture of sea-salt, concentrated sulphuric acid and alcohol, from which it was never thought that any thing else than sulphuric ether could be extracted. He obtained none with the muriatic acid alone. In short, M. Gehlen recognised most of the properties which I did in the muriatic ether. Thus he saw that this ether is most frequently in the state of gas, that it liquefies at about + 10° of Reaumur, that it is slightly soluble in water, that it has a saccharine taste, that it does not redden turnsole tincture, that it does not precipitate the nitrate of silver, and that when we burn it, a great quantity of muriatic acid is developed. M. Gehlen made no experiment, however, to prove from whence this muriatic acid came, or to ascertain the quantity produced by the etherized gas; neither did he attempt to establish the theory of this etherification. It is in this respect only that my labours differ from his. We differ a little also, but not remarkably, as to the process I employed for making the muriatic ether, and by means of which I obtained in an instant probably more ether than by any other, and an ether purer also than M. Gehlen's, since his weighed 845 only, and mine 874, a greater specific gravity in the present instance being a proof of greater purity.

Having no doubt, from the above, that the muriatic ether had been made in Germany, and that its property of producing muriatic acid when burning was also known; and convinced as I was on the other hand, that in France and Spain the fact was unknown, I endeavoured to ascertain if the English chemists knew any thing of the matter. For this purpose, I applied to M. Riffault, the superintendant of the gunpowder manufactories, and who was at that moment translating the third edition of Thomson's System of Chemistry, a work full of erudition, and begun long after M. Gehlen's memoir appeared. M. Riffault read to me every thing respecting the muriatic ether, but I found nothing about Gehlen, or the singular properties he relates of the muriatic ether. Mr. Thomson only speaks of the process of Basse, which consists in mixing melted sea-salt, alcohol, and sulphuric acid; and which, with the exception of the fusion of the salt, has been pointed out by several chemists. I think myself therefore entitled to conclude, that in Great Britain, as well as in France and Spain, the muriatic ether was unknown, and that, being ignorant of M. Gehlen's labours, I have at least the merit of publishing it. How often does it happen that a discovery is made in one country, which had been known in another long before! and this happens because all learned men do not speak one language, and their works are not always translated. This is the case with M. Gehlen's discovery.*

* Since writing the above, M. Boullay, a respectable apothecary in Paris, informed me that he made the ether in question with the muriatic acid and alcohol long ago, although he never gave publicity to his experiments, because he did not think they were so complete as they ought to have been. I am proud to have an opportunity of doing justice to M. Boullay.

Note by M. Thenard.

